# Confirmation of *Galba truncatula* as an intermediate host snail for *Calicophoron daubneyi* in Great Britain, with evidence of alternative snail species hosting *Fasciola hepatica*

**DOI:** 10.1186/s13071-015-1271-x

**Published:** 2015-12-23

**Authors:** Rhys Aled Jones, Hefin Wyn Williams, Sarah Dalesman, Peter M. Brophy

**Affiliations:** Institute of Biological, Environmental and Rural Sciences (IBERS), Aberystwyth University, Aberystwyth, SY23 3DA UK

**Keywords:** *Calicophoron daubneyi*, *Fasciola hepatica*, *Galba truncatula*, *Radix balthica*, *Potamopyrgus antipodarum*, Paramphistomosis, Fasciolosis, Great Britain

## Abstract

**Background:**

*Fasciola hepatica* is a highly prevalent parasite infecting livestock in Great Britain, while *Calicophoron daubneyi* is an emerging parasite within the GB livestock industry. Both *F. hepatica* and *C. daubneyi* require an intermediate host snail to complete their life-cycles and infect ruminants; however, there has been no confirmation of the intermediate host of *C. daubneyi* in GB, while there are questions regarding alternative host snails to *Galba truncatula* for *F. hepatica*. In this study, PCR was used to identify *C. daubneyi* hosting snail species on Welsh pastures and to identify any alternative snail species hosting *F. hepatica.*

**Findings:**

Two hundred and sixty four snails were collected between May-September 2015 from six farms in mid-Wales known to have livestock infected with *C. daubneyi* and *F. hepatica*. Fifteen out of 134 *G. truncatula* were found positive for *C. daubneyi*, one of which was also positive for *F. hepatica*. Three snail species were found positive for *F. hepatica* [18/134 *G. truncatula*, 13/52 *Radix balthica*, and 3/78 *Potamopyrgus antipodarum* (New Zealand mud snail)], but no evidence of *C. daubneyi* infection in the latter two species was found.

**Conclusion:**

This study indicates that *G. truncatula* is a host for *C. daubneyi* in GB. *Galba truncatula* is also an established host of *F. hepatica*, and interactions between both species at intermediate host level could potentially occur. *Radix balthica* and *P. antipodarum* were found positive for *F. hepatica* but not *C. daubneyi*. This could indicate a role for alternative snail species other than *G. truncatula* in infecting pastures with *F. hepatica* in GB.

**Electronic supplementary material:**

The online version of this article (doi:10.1186/s13071-015-1271-x) contains supplementary material, which is available to authorized users.

## Background

Liver fluke (*Fasciola hepatica*) and rumen flukes (Paramphistomatidae spp.) are parasitic trematodes prevalent in GB livestock. Liver fluke disease (Fasciolosis) causes an estimated yearly loss of £300 million for the UK agriculture industry [1], with a study showing that 76 % of the dairy herds in England and Wales are infected [2]. Despite rumen flukes being present in GB for at least half a century [3], it is only in the past decade that these have been regarded as potentially pathogenic parasites, with increasing reports of disease (paramphistomosis) occurrence [4]. This increase may be due to the establishment of *Calicophoron daubneyi* as the prominent paramphistome species in GB, replacing *Paramphistomum cerv*i [5]. How *C. daubneyi* arrived and why it spread across GB has not been confirmed, but increasing animal movements from mainland Europe, where *C. daubneyi* has been present for decades [6], and/or climate change may have facilitated its recent appearance as a parasite of significance.

Both *F. hepatica* and *C. daubneyi* require a snail as an intermediate host in order to complete their life-cycle, a process in which the parasites exploit their host to develop and multiply rapidly. The main intermediate host of *F. hepatica* in GB is *Galba truncatula* (O. F. Müller) [7], however, reports from other countries in Europe have shown that other snail species such as *Radix* spp. [8, 9], *Succinidea* spp. [8], *Omphiscola glabra* (O. F. Müller) [10], and *Lymnaea palustris* (O. F. Müller) [9] can also act as intermediate hosts for *F. hepatica*. Nevertheless, fundamental questions remain regarding the capabilities of these species to support the development of *F. hepatica* from mother sporocyst to cercariae released into the natural environment. In GB, there has been no confirmation of the intermediate snail host of *C. daubneyi. Galba truncatula* has been shown to be the prominent host of *C. daubneyi* in Spain [11] and France, where *O. glabra*, *L. palustris*, *Physa acuta* (Draparnaud), and *R. balthica* (L.) have also been shown to host *C. daubneyi* [12]. Other paramphistome species such as *P. cervi* and *C. calicophoron* are known to infect aquatic snails of the family Planorbidae [13] which are present in the freshwater ecosystems of GB.

This lack of clarity regarding the intermediate host of *C. daubneyi* in GB may have a negative impact on farmers and veterinarians who wish to implement grazing strategies to reduce the burden of *C. daubneyi* in their livestock. There are also questions regarding alternative host species for *F. hepatica* in GB, including whether any shed significant numbers of cercariae onto pasture. In this case study, a panel of snail species found on pastures grazed by ruminants infected with both *F. hepatica* and *C. daubneyi* were screened using PCR assay to detect the presence of infection with these parasites in potential intermediate host snails. The goal was to reveal any *C. daubneyi* transmitting snail species on Welsh pastures and to identify any alternative snail species hosting *F. hepatica*.

## Methods

Between May and September 2015, snails were collected from habitats grazed by animals identified as *C. daubneyi* and *F. hepatica* infected via sedimentation faecal egg count (FEC), using the 10 m transect method [14]. Collected snails were stored in 50 ml tubes and transported to the laboratory where they were identified using morphological characteristics [15]. The snail nomenclature used here follows Anderson [16]. Snails were placed in individual 0.5 ml tubes and crushed using a pellet mixer. Snail DNA was extracted using the Chelex® method [17], adapted with the inclusion of 20 μl of proteinase K (20 mg/ml, Fisher Scientific, Waltham, USA) prior to incubation at 56 °C. After extraction the sample was centrifuged at 15,000 rpm for 6 min with the supernatant collected and diluted ×10. Polymerase chain reaction (PCR) amplification was used to screen snails for infection of *F. hepatica* or/and *C. daubneyi* (Additional file 1: Table S1). In brief, snail DNA of the same species were pooled into groups of six, with each pool subjected to PCR on three occasions to detect *C. daubneyi* infection using primers to amplify a 167 bp strand from the cytochrome *c* oxidase subunit 1 (*cox*1) gene (GenBank JQ815200) and *F. hepatica* infection detected using primers to amplify 425 bp strand from the *cox*1 gene (GenBank AF216697) [11], and finally as a control amplifying 687 bp and 329 bp amplicons of Lymnaeidae spp. and *Potamopyrgus antipodarum* (J. E. Gray) 18S rRNA gene, respectively. Snails from positive groups were screened individually in identical manner to detect infection status. A subset of *C. daubneyi* and *F. hepatica cox*1 gene amplicons detected in infected snails, 18S gene amplicons for *G. truncatula* and *P. antipodarum*, and ITS2 amplicons for *R. balthica* (116 bp amplicon amplified using PCR for *Radix* species ID only), (Additional file 1: Table S1) were sequenced (ABI3100) and aligned to confirm species identity (Geneious Biomatters LTD).

## Results

One hundred and thirty-four *G. truncatula* were sampled from six farms known to have animals infected with both fluke species (referred to as farms 1–6; Table 1). In total 15 were positive for *C. daubneyi*, and 18 were positive for *F. hepatica*. One *G. truncatula* was found positive for *C. daubneyi* and *F. hepatica*. A subset of *C. daubneyi* amplicons (*n* = 5) from positive *G. truncatula* were sequenced and aligned with the *C. daubneyi cox*1 sequence (GenBank JQ815200), and showed 100 % similarity (Additional file 2: Figure S1). Fifty-two *R. balthica* and 78 *P. antipodarum* were collected from farm 5, with *F. hepatica* DNA detected in 13 and 3 snails, respectively, but *C. daubneyi* DNA was not found in either species. A subset of *F. hepatica* amplicons from positive *G. truncatula* (*n* = 2), *R. balthica* (*n* = 1) and *P. antipodarum* (*n* = 1) were sequenced and aligned with the *F. hepatica cox*1 sequence (GenBank AF216697) and showed >99 % similarity (Additional file 2: Figure S2). A subset of *F. hepatica*-positive *G. truncatula* (*n* = 2) and *P. antipodarum* (*n* = 2) were sequenced and aligned with their respective 18S gene sequences (GenBank Z73985.1 and JF960455.1, respectively) with all showing 100 % similarity. *Radix balthica* (*n* = 2) were sequenced and aligned with their ITS2 sequences (GenBank AJ319633.1), and showed 100 % similarity.

**Table 1:** Prevalence of *Calicophoron daubneyi* and *Fasciola hepatica* in *Galba truncatula* collected from six farms in Wales, Great Britain

Farm No.	Month of sampling	*G. truncatula* No. of collected snails	*C. daubneyi* No. of infected snails	*F. hepatica* No. of infected snails	No. of co-infected snails
Farm 1	May	35	0	10	0
Farm 2	June	1	1	0	0
Farm 3	June	24	1	3	0
Farm 4	July	28	0	2	0
Farm 5	August	26	12	1	1
Farm 6	September	20	1	2	0
Total		134	15	18	1

## Discussion

With *C. daubneyi* establishing as a prominent parasite within GB’s livestock industry, further information is required on its epidemiology to allow veterinarians and livestock producers to implement strategies to minimise its impact. This farm survey found that *G. truncatula* is a host for *C. daubneyi* in Wales, which reflects the situation in mainland Europe. If *C. daubneyi* was introduced to GB during the past decade from animals imported from mainland Europe, the fact that its intermediate host *G. truncatula* is abundant in GB is likely to have facilitated its establishment. *Galba truncatula* is already the prominent intermediate host of *F. hepatica* in GB [7]; however, it has been shown in Europe that other lymnaeid snail species can be infected with *F. hepatica*. It is unclear to what extent *F. hepatica* develops in the latter species and whether they may shed significant numbers of cercariae onto pastures. By dissecting a subset of *R. balthica* in our study, free cercariae were seen in snails which were later shown to be positive for *F. hepatica* infection. This would suggest that not only are alternative snail species in GB being infected by *F. hepatica*, but are also shedding cercariae, which could be significant on pastures where *G. truncatula* are absent [8]. Despite the difficulties recorded in experimental infections of *Radix* spp. with *F. hepatica* [18], studies have shown that snails infected at the juvenile stage [19] or persistently exposed to *F. hepatica* over successive generations, are more susceptible to infection and eventual shedding [20]. These results could also explain the *F. hepatica*-positive *P. antipodarum* recorded in our study, however, it must be stressed that these infected snails were not dissected, and thus no confirmation of the patency of infection can currently be made.

With *F. hepatica* and *C. daubneyi* now both present in GB there are unanswered questions regarding potential interactions between these parasites within their intermediate snail hosts. There is evidence to suggest that the presence of *C. daubneyi* within a snail population may facilitate infections with *F. hepatica* [21]; this could increase the susceptibility of alternate snail species to *F. hepatica*. However, co-infections with both parasites in *G. truncatula*, as seen in only one case in this study, have been shown to be rare [22]. This could be down to numerous factors including competition between the two digenean species. *Fasciola hepatica* has been shown to eliminate *C. daubneyi* within *G. truncatula* [23] and it has been suggested that co-infected *G. truncatula* suffer from increased mortality [24]. These two mechanisms could lead to a wide scale antagonism, where the presence of one digenean within a snail population supresses another. This has been hypothesised to be the reason for the absence of *F. hepatica* in populations of *G. truncatula* infected with *Haplometra cylindracea* [24, 25], and species of the Echinostomatidae [26]. Co-infections with *C. daubneyi* and *F. hepatica* have been successfully sustained to cercarial shedding within laboratory settings [23], while a high prevalence of digenean infection within a snail population is required for significant antagonism to occur [27]. Therefore, it could be disputed if any significant antagonism occurs between these two species.

## Conclusion

Our study confirms for the first time that *C. daubneyi* is infecting *G. truncatula* in GB. With a high density of grazing ruminants, widespread populations of *G. truncatula*, endemic *F. hepatica* levels, newly established *C. daubneyi*, and favourable climate for both parasite and intermediate host, a situation may now arise in GB where significant interaction between *F. hepatica* and *C. daubneyi* occurs at intermediate host level. This could in theory impact positively or negatively on the number of viable cercariae shed on pastures due to a synergistic or antagonistic effect; however, it is unclear if this potential interaction would have any major effect on the prevalence of these parasites in livestock. The role of alternative intermediate host snails for *F. hepatica* and *C. daubneyi* should also not be underestimated, with our data concurring with other studies that *F. hepatica* is adaptable in infecting and developing in these species. Further research is required on the intermediate hosts of both *C. daubneyi* and *F. hepatica* and any potential interaction within GB, encompassing greater numbers of snails within a greater extent of snail habitats and farms across a longer period of time.

## Additional files

Additional file 1: Table S1. Primers and PCR cycling conditions used for detection of snail infection status and confirmation of snail identification to the species level (DOCX 16 kb) Additional file 2:
***Calicophoron daubneyi*** **and** ***Fasciola hepatica*** **sequences amplified from infected snails and aligned with GenBank sequences.** **Figure S1.** Sequences for *Calicophoron daubneyi* from infected *Galba truncatula* in farm 1 (GT CD 1), farm 2 (GT CD 4), farm 5 (GT CD 2, GT CD 3), farm 6 (GT CD 5) aligned with *C. daubneyi cox*1 gene sequence (GenBank JQ815200.1). **Figure S2.** Sequences for *Fasciola hepatica* from *Galba truncatula* co-infected with *Calicophoron daubneyi* (GT 2 FH), *Potamopyrgus antipodarum* (PA 1 FH) and *Radix balthica* (RB 1 FH; RB2 FH) aligned with *F. hepatica* cox1 gene sequence (GenBank AF216697.1). (DOCX 17 kb)
