# Supplementary material for: Confirmation of Galba truncatula as an intermediate host snail for Calicophoron daubneyi in Great Britain, with evidence of alternative snail species hosting Fasciola hepatica
Source: Parasit Vectors. 2015 Dec 23;8:656. doi: 10.1186/s13071-015-1271-x (PMC4688931; doi:10.1186/s13071-015-1271-x)
Supplement: Additional file 1: Table S1. — Primers and PCR cycling conditions used for detection of snail infection status and confirmation of snail identification to the species level (DOCX 16 kb) [file 13071_2015_1271_MOESM1_ESM.docx]

**Additional file 1:** **Table S1.** Primers and PCR cycling conditions used for detection of snail infection status and confirmation of snail

| **Target** | **Forward primer** | **Reverse primer** | **Amplicon size** | **Designed by:** | **Initial denaturation** | | **Cycles** | | **Denaturation** | | **Annealing** | | **Extension** | | **Final extension** | |
| --- | --- | --- | --- | --- | --- | --- | --- | --- | --- | --- | --- | --- | --- | --- | --- | --- |
|  |  |  |  |  | Temp °C | Time (m) | |  | Temp °C | Time (s) | Temp °C | Time (s) | Temp °C | Time (s) | Temp °C | Time (m) |
| *C. daubneyi* *cox*1 | GTTTGTGTGGTTTGCCACGG | CTACCCCAAGCAGCCACTAC | 167 | This study | 95 | 10 | | 40 | 95 | 30 | 63 | 30 | 72 | 45 | 72 | 10 |
| *F. hepatica* *cox*1 | GCCGGGTCCTCAACATAATA | AGCACAAAATCCTGATCTTACCA | 425 | [1] | 95 | 10 | | 40 | 95 | 30 | 63 | 30 | 72 | 45 | 72 | 10 |
| Lymnaeidae spp. 18S* | CTGGTTGATCCTGCCAGT | CAGACAAGTAGGGCAGGATTT | 687 | This study | 95 | 10 | | 35 | 95 | 30 | 65 | 30 | 72 | 60 | 72 | 10 |
| *Potamopyrgus* sp. 18S | AACAGCTCCGACCCTCAC | CCTCAAATGAGTTCCGTATTGT | 329 | This study | 95 | 10 | | 35 | 95 | 30 | 60 | 30 | 72 | 30 | 72 | 10 |
| *Radix sp*. ITS2** | CGCGCTCTGGWCCKTCGCGGC | CGGACAACGGCGCGGCTTG | 116 | [2] | 95 | 10 | | 35 | 95 | 30 | 65 | 30 | 72 | 30 | 72 | 10 |

* *Lymnaeidae* spp. 18S primers were used in PCR as controls for *G. truncatula* and *R. balthica* snails and for sequencing of *G. truncatula ** Radix* sp. ITS2 primers were used to sequence *R. balthica*

**References**

1. Martinez-Ibeas AM, Gonzalez-Warleta M, Martinez-Valladares M, Castro-Hermida JA, Gonzalez-Lanza C, Minambres B et al. **Development and validation of a mtDNA multiplex PCR for identification and discrimination of *Calicophoron daubneyi* and *Fasciola hepatica* in the *Galba truncatula* snail**. *Vet Parasitol.* 2013;**195(1-2)**:57-64. doi:10.1016/j.vetpar.2012.12.048.

2. Jouet D, Ferte H, Depaquit J, Rudolfova J, Latour P, Zanella D et al. ***Trichobilharzia* spp. in natural conditions in Annecy Lake, France**. *Parasitol Res.* 2008;**103(1)**:51-8. doi:10.1007/s00436-008-0926-3.
