# Supplementary material for: Confirmation of Galba truncatula as an intermediate host snail for Calicophoron daubneyi in Great Britain, with evidence of alternative snail species hosting Fasciola hepatica
Source: Parasit Vectors. 2015 Dec 23;8:656. doi: 10.1186/s13071-015-1271-x (PMC4688931; doi:10.1186/s13071-015-1271-x)
Supplement: Additional file 2: — Calicophoron daubneyi and Fasciola hepatica sequences amplified from infected snails and aligned with GenBank sequences. Figure S1. Sequences for Calicophoron daubneyi from infected Galba truncatula in farm 1 (GT CD 1), farm 2 (GT CD 4), farm 5 (GT CD 2, GT CD 3), farm 6 (GT CD 5) aligned with C. daubneyi cox1 gene sequence (GenBank JQ815200.1). Figure S2. Sequences for Fasciola hepatica from Galba truncatula co-infected with Calicophoron daubneyi (GT 2 FH), Potamopyrgus antipodarum (PA 1 FH) and Radix balthica (RB 1 FH; RB2 FH) aligned with F. hepatica cox1 gene sequence (GenBank AF216697.1). (DOCX 17 kb) [file 13071_2015_1271_MOESM2_ESM.docx]

**Additional file 2:** ***Calicophoron daubneyi* and *Fasciola hepatica* sequences amplified from infected snails and aligned with GenBank sequences. Figures S1 – S2.**

1 10 20 30 40 50 60

| | | | | | |

GT CD 1 ------------TTTGTGTGGTTTGCCACGGCGTGTTTGTGTTTATAATCCTGATTTTTT

GT CD 2 ----------------------------------GTTTGTGTTTATAATCCTGATTTTTT

GT CD 3 -----------GTTTGTGTGGTTTGCCACGGCGTGTTTGTGTTTATAATCCTGATTTTTT

GT CD 4 ------------------------------------------------------------

GT CD 5 -----------GTTTGTGTGGTTTGCCACGGCGTGTTTGTGTTTATAATCCTGATTTTTT

JQ815200.1 GCACTATTTYGGTTTGTGTGGTTTGCCACGGCGTGTTTGTGTTTATAATCCTGATTTTTT

GT CD 1 TTGGTTGGAGAGTTTGGCGTCTTTTGGTGCTTTTTTGTCGGTGATTAGTGCTTTTTTTTT

GT CD 2 TTGGTTGGAGAGTTTGGCGTCTTTTGGTGCTTTTTTGTCGGTGATTAGTGCTTTTTTTTT

GT CD 3 TTGGTTGGAGAGTTTGGCGTCTTTTGGTGCTTTTTTGTCGGTGATTAGTGCTTTTTTTTT

GT CD 4 -----TGGAGAGTTTGGCGTCTTTTGGTGCTTTTTTGTCGGTGATTAGTGCTTTTTTTTT

GT CD 5 TTGGTTGGAGAGTTTGGCGTCTTTTGGTGCTTTTTTGTCGGTGATTAGTGCTTTTTTTTT

JQ815200.1 TTGGTTGGAGAGTTTGGCGTCTTTTGGTGCTTTTTTGTCGGTGATTAGTGCTTTTTTTTT

GT CD 1 GGTAC-------------------------------------------------------

GT CD 2 GGTACTTATTTTGTGGGAGTCTTTGGTTGTACATAATGTTGTAGTGGCTGCTTGGGGTAG

GT CD 3 GGTACTTATTTTGTGGGAGTCTTTGGTTGTACATAATGTTGTAGTGGCTGCTTGGGGTAG

GT CD 4 GGTACTTATTTTGTGGGAGTCTTTGGTTGTACATAATGTTGTAGTGGCTGCTTGGGGTAG

GT CD 5 GGTACTTATTTTGTGGGAGTCTTTGGTTGTACATAATGTTGTAGTGGCTGCTTGGGGTAG

JQ815200.1 GGTACTTATTTTGTGGGAGTCTTTGGTTGTACATAATGTTGTAGTGGCTGCTTGGGGTAG

**Figure S1.** Sequences for *Calicophoron daubneyi* from infected *Galba truncatula* in farm 1 (GT CD 1), farm 2 (GT CD 4), farm 5 (GT CD 2, GT CD 3), farm 6 (GT CD 5) aligned with *C. daubneyi cox*1 gene sequence (GenBank JQ815200.1).

1 10 20 30 40 50 60

| | | | | | |

AF216697.1 GCCAGGTCCTCAACATAATAGTTATATGAATGGTGTTGGTCGCTGGGTTTTTTAGTTGTT

GT 2 FH -----------------------------------------------------AGTTGTT

PA 1 FH GCC**G**GGTCCTCAACATAATAGTTATATGAATGGTGTTGGTCGCTGGGTTTTTTAGTTGTT

RB 1 FH -------CCTCAACATAATAGTTATATGAATGGTGTTGGTCGCTGGGTTTTTTAGTTGTT

RB 2 FH -----------------------------------------------------AGTTGTT

AF216697.1 TGGGGTTTGTTGGGGGGTTAGTTTAGGTATTTTTAGAATTCTGCTTTTGTAAAGCAGAGG

GT 2 FH TGGGGTTTGTTGGGGGGTTAGTTTAGGTATTTTTAGAATTCTGCTTTTGTAAAGCAGAGG

PA 1 FH TGGGGTTTGTTGGGGGGTTAGTTTAGGTATTTTTAGAATTCTGCTTTTGTAAAGCAGAGG

RB 1 FH TGGGGTTTGTTGGGGGGTTAGTTTAGGTATTTTTAGAATTCTGCTTTTGTAAAGCAGAGG

RB 2 FH TGGGGTTTGTTGGGGGGTTAGTTTAGGTATTTTTAGAATTCTGCTTTTGTAAAGCAGAGG

AF216697.1 TGGTTTTTGGCTGCTCTCTATGTTTTATTGGCTGTGAGGTTGATTTTATTTGGGTTTCGT

GT 2 FH TGGTTTTTGGCTGCTCTCTATGTTTTATTGGCTGTGAGGTTGATTTTATTTGGGTTTCGT

PA 1 FH TGGTTTTTGGCTGCTCTCTATGTTTTATTGGCTGTGAGGTTGATTTTATTTGGGTTTCGT

RB 1 FH TGGTTTTTGGCTGCTCTCTATGTTTTATTGGCTGTGAGGTTGATTTTATTTGGGTTTCGT

RB 2 FH TGGTTTTTGGCTGCTCTCTATGTTTTATTGGCTGTGAGGTTGATTTTATTTGGGTTTCGT

AF216697.1 TTTTTAGATTACCTTTTGCATCATGATTTGTTGATTTTTGGTTTGGATTGATTGTTTCCG

GT 2 FH TTTTTAGATTACCTTTTGCATCATGATTTGTTGATTTTTGGTTTGGATTGATTGTTTCCG

PA 1 FH TTTTTAGATTACCTTTTGCATCATGATTTGTTGATTTTTGGTTTGGATTGATTGTTTCCG

RB 1 FH TTTTTAGATTACCTTTTGCATCATGATTTGTTGATTTTTGGTTTGGATTGATTGTTTCCG

RB 2 FH TTTTTAGATTACCTTTTGCATCATGATTTGTTGATTTTTGGTTTGGATTGATTGTTTCCG

AF216697.1 AAGAGTACTGATTTTTGTTTTGCTTGCTTAGGGTTGTTTTGGAGGTGAGTAACTTTTTGT

GT 2 FH AAGAGTACTGATTTTTGTTTTGCTTGCTTAGGGTTGTTTTGGAGGTGAGTAACTTTTTGT

PA 1 FH AAGAGTACTGATTTTTGTTTTGCTTGCTTAGGGTTGTTTTGGAGGTGAGTAACTTTTTGT

RB 1 FH AAGAGTACTGATTTTTGTTTTGCTTGCTTAGGGTTGTTTTGGAGGTGAGTAACTTTTTGT

RB 2 FH AAGAGTACTGATTTTTGTTTTGCTTGCTTAGGGTTGTTTTGGAGGTGAGTAACTTTTTGT

AF216697.1 AGAGCTTAATTGAGGTTGTGATAGGTGATTCGGAGTACTTGATATCTGATTTTGAGTAGA

GT 2 FH AGAGCTTAATTGAGGTTGTGATAGGTGATTCGGAGTACTTGATATCTGATTTTGAGT---

PA 1 FH AGAGCTTAATTGAGGTTGTGATAGGTGATTCGGAGTACTTGATATCTGATTTTGAGTAGA

RB 1 FH AGAGCTTAATTGAGGTTGTGATAGGTGATTCGGAGTACTTGATATCTGATTTTGAGTAGA

RB 2 FH AGAGCTTAATTGAGGTTGTGATAGGTGATTCGGAGTACTTGATATCTGATTTTGAGTAGA

AF216697.1 TTTTGTTGGCATATTGCGGCTTAGTTATGATATTAGGTCGTTGGTAAGATCAGGATTTTG

GT 2 FH ------------------------------------------------------------

PA 1 FH TTTTGTTGG**T**ATATTGCGGCTTAGTTATGATATTAGGTCGTTGGTAAGATCAGGATTTTG

RB 1 FH TTTTGTTGGCATATTGCGGCTTAGTTATGATATTAGGTCGTTGGTAAGATCAGGATTTTG

RB 2 FH TTTTGTTGGCATATTGCGGCTTAGTTATGATATTAGGTCGTTGGTAAGATCAGGATTTTG

AF216697.1 TGCTTTTTGTAATAGGGATTAAAATTCTCCTGATTTTAGGTGTGTGTTATTATTCGTTTG

GT 2 FH ------------------------------------------------------------

PA 1 FH TGCT--------------------------------------------------------

RB 1 FH TGCT--------------------------------------------------------

RB 2 FH TGC---------------------------------------------------------

**Figure S2.** Sequences for *Fasciola hepatica* from *Galba truncatula* co-infected with *Calicophoron daubneyi* (GT 2 FH), *Potamopyrgus antipodarum* (PA 1 FH) and *Radix balthica* (RB 1 FH; RB2 FH) aligned with *F. hepatica* cox1 gene sequence (GenBank AF216697.1).
